# Design and Implementation of a YARP Device Driver Interface: The Depth-Sensor Case

**DOI:** 10.3389/frobt.2018.00040

**Published:** 2018-04-09

**Authors:** Alberto Cardellino, A. Ruzzenenti, L. Natale

**Affiliations:** iCub Facility, Istituto Italiano di Tecnologia, Genoa, Italy

**Keywords:** hardware abstraction, client server architecture, software design, depth sensor, YARP

## Abstract

This work illustrates the design phases leading to the development of a new YARP device interface along with its client/server implementation. In order to obtain a smoother integration and a more reliable software usability, while avoiding common errors during the design phases, a new interface is created in the YARP network when a new family of devices is introduced.

## 1. Introduction

Depth sensors, such as the kinect (Zhang, 2012; Han et al., 2013), are very popular in the field of navigation for mobile robots. OpenNI2 framework (Aksoy et al., 2011; Rehem Neto et al., 2013), an open source SDK used for the development of 3D sensing middleware libraries and applications, is arising as a tentative standard for this type of devices, yet producers do not always comply with the specifications. In a typical application, data are acquired by a robot but processed and visualized on a remote machine. The device driver is in charge of acquiring data from the sensor while client and server handles the transfer, optimizing both portability and performance. In general terms, we believe that an effective solution to standardize data flow in a software framework is to provide the device interface together with its client/server implementation.

### 1.1. YARP device interface

YARP (Metta et al., 2006) is a middleware specifically designed for robotics with a strong focus on modularity, code re-usage, flexibility, and hw/sw abstraction. In order to achieve those goals, the use of interfaces is fundamental because they allow to abstract from a specific producer. YARP device driver interfaces are the ones devoted to generalize the capabilities and configurations of a specific set of similar devices.

**Figure 1:**
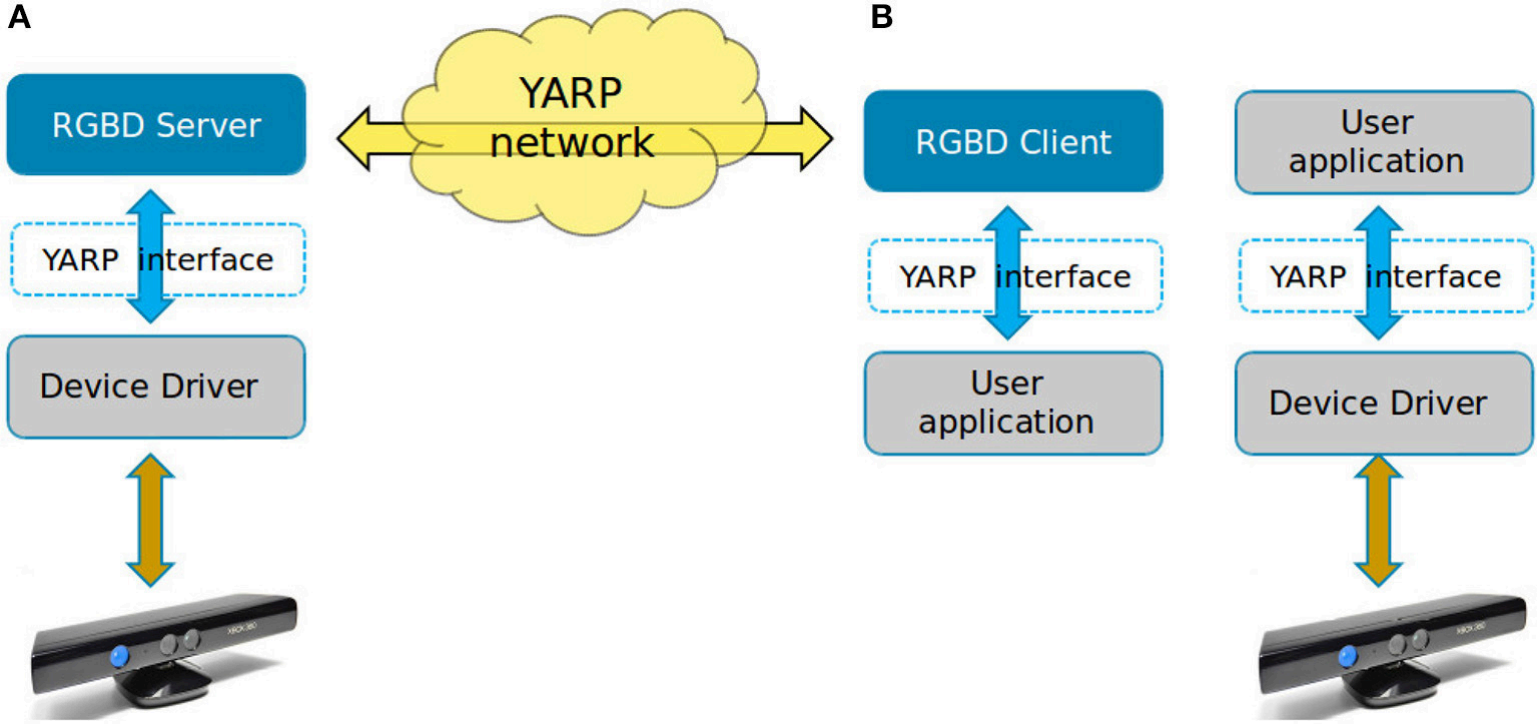
How the application connects to an hardware device. **(A)** Via client/server. **(B)** Directly.

An interface is a class composed only by pure virtual function, data type definitions, and it is the place where relevant measurement unit must be declared. The implementation of a new YARP device interface is realized in the development of three C++ objects: (i) the device driver which handles the real hardware, (ii) the network server which publish the data, and (iii) the network client used by the application to remotely access the device. The objects are shown in the Figure 1A.

Note that by mean of an interface, the user application can connect directly to the device driver bypassing the client/server architecture, as illustrated in Figure 1B. This is useful when higher efficiency and low latency are required.

### 1.2. RGBD device family

An RGBD sensor is a device equipped with a standard RGB color camera and a depth image source. The latter is producing a special image in which each pixel is providing the distance of closest object in view.

RGBD is the source data required to build a point cloud, but they have distinct characteristics. The depth sensor produces two separated image frames where the first one contains color component and the second one distance information. A point cloud instead is a specific data type where the *point* contains color and depth components altogether and optionally other related information like surface normals, curvatures, histogram, and so on.

While both RGB and depth frames shared the rectangular *width per height* structure, a point cloud is an unordered list of points of any size and shape. When dealing with this type of sensors, a number of information is required in order to correctly extract valuable data. Besides the image dimensions in terms of pixels and the frames themselves, other useful parameters are, the lens distortion model of RGB cameras and the measurement range and its accuracy for the depth sensor. The designing of the interface should thus include and provide all the previously mentioned data.

**Figure 2:**
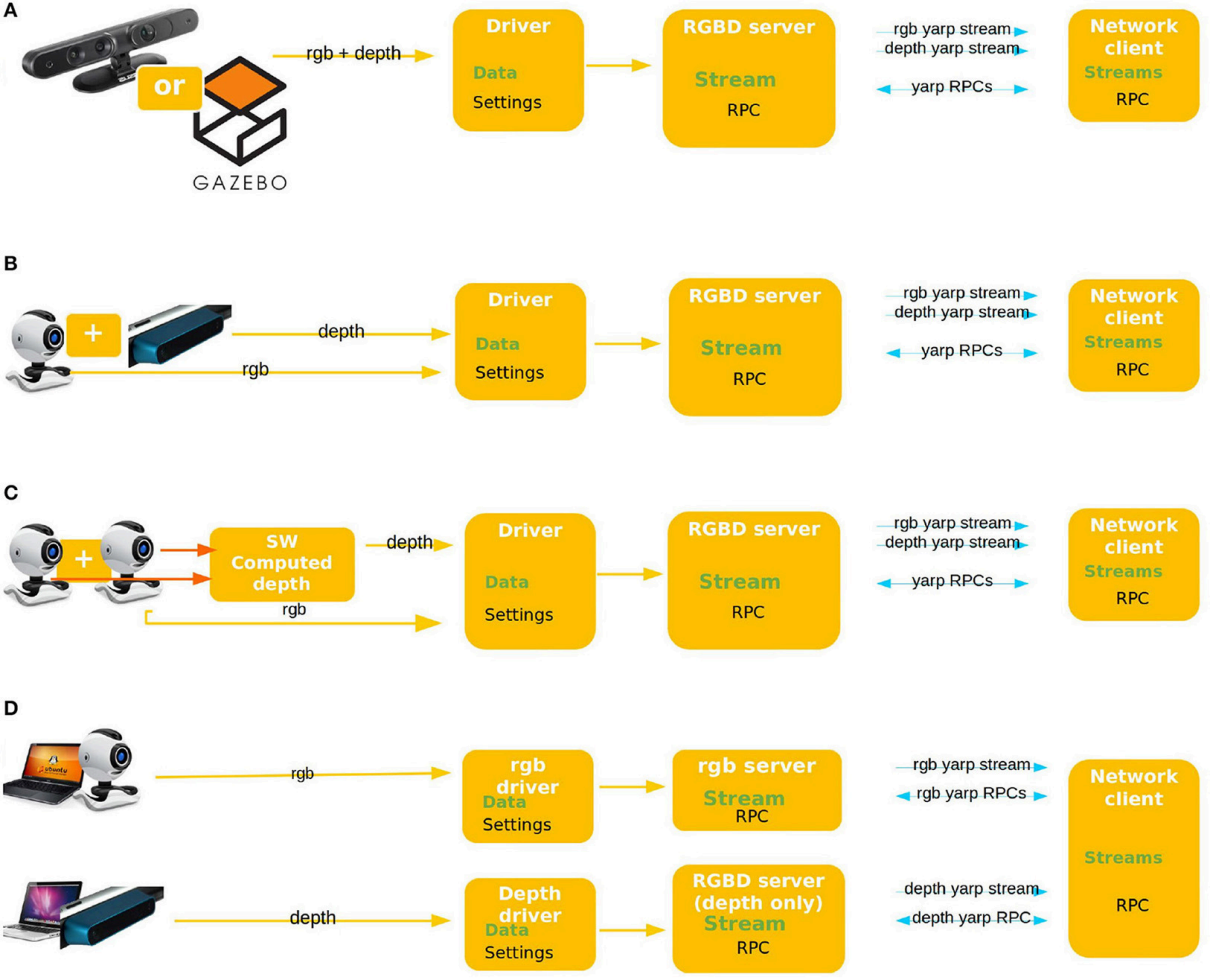
Use case scenarios. **(A)** Proper RGBD sensor: MicroSoft Kinect, Asus XTION, or similar. **(B)** Two local yet separated sensors. **(C)** Stereo vision: two RGB sensor and a computed depth image. **(D)** Two separated sensors physically connected to two different machines.

In this work, the concept of RGBD device is extended to include more cases than the physical sensor. All cases are shown in the Figure 2.

### 1.3. Common design patterns

The quickest approach for designing an Hardware Abstraction Layer (HAL) is proceeding bottom-up, starting from the hardware capabilities and generalizing them. This approach tends to fail when the device generates non-standard data or when the underlying hardware varies significantly from sample to sample.

On one hand, bottom-up generated interfaces are comprehensive of all device features whilst, on the other hand those interfaces tends to be too tailored on the first device they were built upon and difficult to be reused when the underlying assumptions change.

The other most followed lead is the top-down approach which is capable of providing a better abstraction, but usually it fails being comprehensive. In this case, low level details or configurations may be missing and users do not have access to all the required data.

## 2. Design process

An example of well-structured software design is illustrated in the NEPOMUK project paper (Groza et al., 2007), described as an iterative process starting from the user's need, to design new code in order to seamlessly fit into an existing software environment. In order to overcome the before mentioned limitations, the design process has been widened to a bigger-picture, real use cases have been analyzed and generalized to extract relevant functionalities. The latter information has been employed to analyze the data flow and to design the resulting interface.

### 2.1. Identifying data flow and device capabilities

Typically, an RGBD device is capable of producing a color image and a depth image along with their respective parameters. The interfaces are thus required to describe and provide equivalent streams and parameters. YARP is often referred to as the robot information piping system, because one of its main functions is exchanging data between applications. Identifying the data to be shared and their properties helps designing better interfaces and client/server objects.

There are two main ways to exchange data in YARP, called streaming and RPC. All the information the device streams are sent to the client, whereas all the get/set requests originated by the client are RPCs. Data are sent trough the network via an object called *port*. A port is an abstraction of the operative system socket and it is able to send any type of data, different protocols can be used and multiple consumer can read from a single producer. We can identify the following desired data:

- Streaming
  ⇒ The RGB image
  ⇒ The depth image
- RPCs
  ⇔ Info about streaming: e.g., how big is the image being published
  ⇔ Controls: e.g., increase the saturation/brightness and other camera parameters.
  ⇔ Info Visual: e.g., get the field of view of RGB/depth camera
  ⇔ Info about HW: e.g., this is a USB device

Each piece of information is required for both RGB and depth separately because they may differ in availability and values. Note that interfaces and data flow do not need to match. For example, a single interface may include both streaming and RPC data while a single RPC connection can handle requests from multiple interfaces.

### 2.2. Identifying use case scenarios

The analysis result in four scenarios being comprehensive for all foreseen real world uses of a RGBD sensor, shown in Figure 2.

Note: Each sensor can be a real or simulated one, they will be handled in the same way.

Among existing applications, yarpview and camCalib are the most important ones the new device has to be compatible with. The first one is a GUI used to display images while the latter is used to compensate lens distortion.

### 2.3. Additional constraints and requirements

It is useful to explicit a few other characteristics the new interface and its implementation shall have in order to cope with the needs of an highly dynamic and innovative field like robotics.

#### 2.3.1. Need of a standard

Different devices may provide the same data using incompatible formats, for example the distance measure can be measured in meters, millimeter, or other units while the binary implementation can be an integer or a floating point number. Furthermore, a lens distortion model can be described using different set of parameters. In order for an high level application to run on different robots, it must be able to get all information at runtime and use them properly.

Relevant settings that are not available from OpenNI API have to be acquired from another source, for example distortion model can rarely be retrieved from the OpenNI API.

#### 2.3.2. Unique traits of rgbd device

This work has to deal with the intrinsic complexity of a device composed by two different sensors with similar characteristics which may be unclear. A large amount of parameters are required to correctly identify the device properties.

#### 2.3.3. Compliance with the YARP ecosystem

This software is part of the YARP middleware, hence the new interface has to fit into existing code and to be as much intuitive as possible for both experienced and novice users. The RGB sensor is by all means a standard camera and, as such, it provides options to configure color properties like saturation, brightness, exposure etc…The ability to change the camera parameters at runtime is widely used, so it must be accessible via the interface. The optimal solution is to allow any software currently working with standard camera to work also with RGB part of this device without any changes.

#### 2.3.4. Modularity

YARP heavily leverages on modularity and code re-use, therefore the implementation of the depth sensor interface has to maximize these best practices. Furthermore, the client can read data from multiple sources while the server can broadcast them independently, as in use cases (Figures 2B,D).

#### 2.3.5. Re-usability

Re-usability check has to be performed in two ways: first looking for compatible code to re-use into this project and second creating code that may be useful outside the scope of RGBD device for future use.

## 3. Adopted solutions

The design process resulted in a series of design considerations and technical solutions adopted to best attain all the requirements. Those solutions can be divided into “abstract” design criteria and their relative “concrete” implementation.

All the requirements and solutions are summarized in the Table 1.

**Table 1:** Requirements and proposed solutions for new YARP interface.

**Requirement**	**Design criteria**	**Implementation solutions**
Need of a standard	Definition of a YARP standard	API compensation
Compatibility with the YARP ecosystem	Re-use, not inherit	Separated data flow
Modularity	Isolation of capabilities	Separated data flows
Re-usability	Isolation of capabilities	Three levels decoupling
Unique traits of this device type	Isolation of capabilities	Capabilities composition

The resulting structure is general enough to cover all the use cases and flexible to allow both incremental implementation and update of existing software. The current state is already able to handle use case scenarios (Figures 2A,C) and can be easily extended to handle also cases (Figures 2B,D).

### 3.1. Design criteria

#### 3.1.1. Definition of a YARP standard

YARP uses international measurement system for all units (except for angular degrees), therefore this convention has been enforced also in this interface where the unit for the depth measurement is set to be meters. The binary representations is the float to allow fraction of meters.

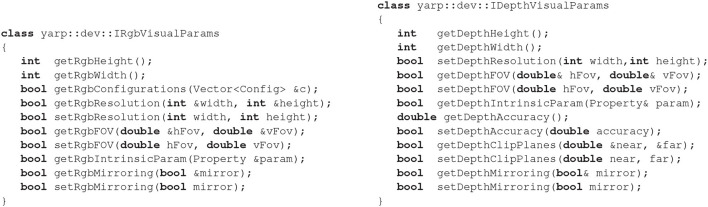

**Interface snapshot 1**. Example of interface methods.
Documentation: http://www.yarp.it/classyarp_1_1dev_1_1IRgbVisualParams.html
http://www.yarp.it/classyarp_1_1dev_1_1IDepthVisualParams.html
Git repository: https://github.com/robotology/yarp/tree/master/src/libYARP_dev/include/yarp/dev

#### 3.1.2. Re-use, not inherit

The interface IFrameGrabberControls2 is an already existing YARP interface describing how to set RGB color sensor properties as saturation, brightness, exposure etc…A possible way to include these functionalities in the new interface would be to inherit from it, but this has some implications. The new interface will be tightly coupled to previous code and the maintenance will be more difficult. Any change to IFrameGrabberControls2 will be propagated to the new interface and all devices using it. On the other hand, adding the same methods also in the new interface will generate duplicated code and confusion.

The best approach is to keep separated the two functionalities and have the device implementation to use them where required.

#### 3.1.3. Isolation of capabilities

Instead of defining an single interface covering all the device functionalities or data types, the best solution is to define an interface for each capability and then combine them into a bigger one where appropriate. This way each interface is smaller and cleaner, but most importantly each single interface can be re-used more easily in different contexts. New interfaces created for this device are the ones required to fill the gap between what's existing and what is required. They have been created separately for RGB and depth part of the device. A snippet of code is shown below.

The two interfaces created are similar because the sensors have similar features, but each method has the RGB/Depth prefix to clearly state which sensor it is working with. This helps novice users to understand what the function is supposed to do and name clash between two sensors is avoided. There are some differences however due to the sensors nature, for example in the depth interface there are getter and setter methods for Accuracy and clip planes which has no meaning for a standard RGB camera.

### 3.2. Implementation solutions

#### 3.2.1. Api compensation

The information requested to be available are more then what's usually covered by the OpenNI2 API, hence another source of information is needed. This has been achieved by mean of a configuration file, subdivided in three main sections:

- General parameters: describe which device the YARP factory shall create and how to manage it.
- Settings: these parameters describe the user's desired initial configuration of the device. These values will be set at startup and if anyone fails, the device must be closed providing an error. All the settings are also available for remote control with getter/setter methods, therefore the configuration can be verified and changed remotely by the user's application at any time.
- Hardware description: the listed parameters are read only. Everything not available through device API can be listed here. These values will be available to remote applications via getter methods, but they cannot be set. This is also useful in case the device returns wrong values; the data from configuration file will be returned to the user instead.

#### 3.2.2. Separated data flow

Defining how many sockets to create, the protocol to use etc…is a trade-off between optimization of resources and granularity of information. The more complex/custom the data is, the less application will be compatible with. On the other hand, creating many sockets to send small pieces of information is a waste of resources.

The choice implemented is to create two separated streams for RGB and depth images, to be back compatible with existing application using color images only. All the RPC requests instead can be handled by a single YARP port. There is no need in fact for the client to know all the server's capabilities. A client can implement only the subset of RPC it requires, therefore a existing client can freely work with a newer server using an extended set of messages.

Only the 4th use case scenario will require the client to have two separated RPC ports, as it requires to connect to two different servers to collect all the required informations.

#### 3.2.3. Three levels decoupling

Network messages, client/server implementation and hardware device are separated between each-others. Usually when building a client/server pair in YARP there are two levels of decoupling: the first one is the YARP message which decouples the server from the remote client. The second one is the interface itself which decouples the server from the device driver and the user application from the network client.

The implementation of an interface in the server/client requires to write code devote to generate the YARP message and parse it in order to provide the service and generate proper response. Historically this job was always been implemented by the client/server classes themselves, but this may lead to duplicated code when more servers or clients uses the same interface. Therefore a new decoupling level has been introduced by implementing all the YARP message parsing into a specific class for each interface, the client/server will then use these classes to handle network communication.

This way, should a new server implement this interface, adding the message parsing will require only three lines of code:

1) Add interface inheritance
Server : public NewInterface
2) Instantiate a parser class
yarp::dev::Implement_Interface_Parser rgbParser;
3) Configure the parser by giving access to the class
rgbParser.configure(NewInterfacePointer);
**Interface snapshot 2**. Example of usage, server side.

#### 3.2.4. Capabilities composition

Leveraging on the previously shown ideas “Isolation of Capabilities” and “Three Levels Decoupling,” it follows that a device can incrementally add capabilities by inheriting from required interfaces and parsers. The whole RGBD interface will be the sum of RgbVisualParams and DepthVisualParams, plus the specific information which have meaning only when both sensors are available together.


**class** yarp::dev::IRGBDSensor : **public** IRgbVisualParams
**public** IDepthVisualParams
{
    **bool** getExtrinsicParam(Matrix &extrinsic);
      string getLastErrorMsg(Stamp *timeStamp);
    **bool** getRgbImage(FlexImage &rgbImage, Stamp*timeStamp);
    **bool** getDepthImage(ImageOf<PixelFloat>
    &depthImage, Stamp *timeStamp);
    **bool** getImages(FlexImage &colorFrame, ImageOf
    <PixelFloat> &depthFrame, Stamp *colorStamp, Stamp *depthStamp);
    RGBDSensor_status getSensorStatus();
}

**Interface snapshot 3**. Extending capabilities by merging two interfaces into a bigger one.
Documentation: http://www.yarp.it/classyarp_1_1dev_1_1IRGBDSensor.html
Git repository: https://github.com/robotology/yarp/blob/master/src/libYARP_dev/include/yarp/dev/IRGBDSensor.h

Each interface contains methods to get the sensor intrinsic parameters, and since the RGBD interface includes the two sensors together, a method to get extrinsic parameters is included. The client/server for this device will create its own message sender/parser by extending the ones implemented for each single interface as explained in “Three Levels Decoupling” section. Furthermore, previous RGB-only image server has been easily extended to implement also the RgbVisualParams interface by adding the parser.

## 4. Conclusion and future work

The design process successfully generated a set of interfaces both flexible and comprehensive to handle all use cases identified and satisfy all additional requirements. The interface and C++ objects shown in this work have been used with three models of depth sensors from two different producers and with the simulated device available within Gazebo. The new server is well-integrated in the YARP framework, compatibility with existing applications has been achieved and former device drivers specific for RGB-only cameras have been extended to implement new functionality, hence user application can benefit from additional information.

The dataset acquisition pipeline shown in Pasquale et al. (2016) and used in Maiettini et al. (2017) was developed for the iCub robot using images acquired from stereo vision system and then, using the interfaces resulting from the work presented, the pipeline was easily integrated on the R1 robot, that mounts a RGBD sensor.

The implementation will be extended to cover also use scenarios (Figures 2B,D), also a synchronization mechanism for the two image streaming will be integrated in the client. The code can be verified using YARP test utilities or using simple example code. Instruction how to run tests are in the in the following github repository https://github.com/robotology/yarp/tree/master/example/dev/RGBD/README.md.

## Author contributions

AC: main contributor, designed the interfaces, and client/server implementation; AR: contributed refining the interfaces, device driver implementation, testing; LN: supervisor.

### Conflict of interest statement

The authors declare that the research was conducted in the absence of any commercial or financial relationships that could be construed as a potential conflict of interest. The reviewer HC, and handling Editor declared their shared affiliation.
